# Chloral for Sea-Sickness

**Published:** 1871-05

**Authors:** 


Chloral for Sea-Sickness.—It has become the fashion for travelers about to cross the British Channel, and who are liable to sea-sickness, to escape the trouble by taking a dose of chloral before starting, and sleeping through the voyage. This will hardly do for crossing the Atlantic, or for the California voyage. But a judicious use of the remedy will at least serve to give the sufferer a sound night's sleep, and protect him from the demon of the sea for half of each twenty-four hours.—*Pacific Medical Journal.*

